# Predicting Dimensional Antidepressant Response to Repetitive Transcranial Magnetic Stimulation using Pretreatment Resting-state Functional Connectivity

**DOI:** 10.21203/rs.3.rs-3204245/v1

**Published:** 2023-08-07

**Authors:** Benjamin Wade, Tracy Barbour, Kristen Ellard, Joan Camprodon

**Affiliations:** Massachusetts General Hospital; Massachusetts General Hospital, Harvard Medical School

## Abstract

Repetitive transcranial magnetic stimulation (rTMS) is an effective treatment for depression and has been shown to modulate resting-state functional connectivity (RSFC) of depression-relevant neural circuits. To date, however, few studies have investigated whether individual treatment-related symptom changes are predictable from pretreatment RSFC. We use machine learning to predict dimensional changes in depressive symptoms using pretreatment patterns of RSFC. We hypothesized that changes in dimensional depressive symptoms would be predicted more accurately than scale total scores. Patients with depression (n=26) underwent pretreatment RSFC MRI. Depressive symptoms were assessed with the 17-item Hamilton Depression Rating Scale (HDRS-17). Random forest regression (RFR) models were trained and tested to predict treatment-related symptom changes captured by the HDRS-17, HDRS-6 and three previously identified HDRS subscales: core mood/anhedonia (CMA), somatic disturbances, and insomnia. Changes along the CMA, HDRS-17, and HDRS-6 were predicted significantly above chance, with 9%, 2%, and 2% of out-of-sample outcome variance explained, respectively (all p<0.01). CMA changes were predicted more accurately than the HDRS-17 (p<0.05). Higher baseline global connectivity (GC) of default mode network (DMN) subregions and the somatomotor network (SMN) predicted poorer symptom reduction, while higher GC of the right dorsal attention (DAN) frontoparietal control (FPCN), and visual networks (VN) predicted reduced CMA symptoms. HDRS-17 and HDRS-6 changes were predicted with similar GC patterns. These results suggest that RSFC spanning the DMN, SMN, DAN, FPCN, and VN subregions predict dimensional changes with greater accuracy than syndromal changes following rTMS. These findings highlight the need to assess more granular clinical dimensions in therapeutic studies, particularly device neuromodulation studies, and echo earlier studies supporting that dimensional outcomes improve model accuracy.

## Introduction

Repetitive transcranial magnetic stimulation (rTMS) administered to the left dorsolateral prefrontal cortex (DLPFC) is a U.S. Food and Drug Administration (FDA) approved treatment for treatment-resistant depression [1-3]. It is a noninvasive and robust antidepressant treatment with response and remission rates ranging from 40–50% and 25–30%, respectively [4, 5]. High-frequency rTMS (≥5Hz) produces long-term potentiation-like excitatory effects on underlying neural populations, leading to circuit-wide neuromodulatory effects [6, 7]. Depression has long been characterized as a network-based disorder involving disrupted connectivity and metabolic activity of the subgenual anterior cingulate (sgACC), hippocampus and DLPFC [8-10]. While modulation of sgACC activity is widely associated with successful antidepressant response [11], rTMS is only capable of directly stimulating superficial cortical regions, though these act as windows that allow transsynaptic distal and indirect modulation of connected nodes. Targeting of the left DLPFC is therefore motivated by its relative accessibility as a more superficial target within this putative depression network, and its downstream connections with nodes relevant to mood disorders: successful rTMS response is thought to be mediated, in part, by increased inhibition of the sgACC through the modulation of the DLPFC [2, 8, 12].

Despite the hypothesized network-level mechanisms of antidepressant response to rTMS, few studies have investigated whether rTMS treatment outcomes are predictable using pretreatment MRI-based resting-state functional connectivity (RSFC) measures. Among these, a recent study investigating RSFC predictors of rTMS in 47 patients reported that regional blood oxygen level dependent (BOLD) signal power and connectivity of the default mode network (DMN) could predict treatment response with 85–95% accuracy [13]. Additional correlative studies have reported that the pretreatment volume of the left superior frontal and caudal middle frontal cortices [4] and thickness of the left rostral ACC [14] are predictive of rTMS response. Another study using accelerated intermittent theta burst stimulation (aiTBS) in 50 patients to treat depression identified a correlation between pretreatment caudal cortical thickness measures ACC and subsequent treatment response [15]. These studies suggest that structural or functional properties of networks and regions commonly affected by depression might be predictive of rTMS response.

The symptomatic heterogeneity of depression is a well-known obstacle to the identification of treatment-predictive biomarkers. Concretely, a DSM-based diagnosis of major depressive disorder (MDD) requires five out of nine symptoms allowing for 227 potential symptom constellations to arrive at the same diagnosis (i.e., equifinality), and even the possibility that 2 patients diagnosed with MDD do not have overlapping symptoms. This varied range of potential symptom constellations may be underpinned by dysfunctions in unique and/or overlapping neural circuits. For example, a review of 59 fMRI studies on patients with current or remitted depression reported that two separable aspects of anhedonia, reward learning and reward liking, are characterized by reduced frontostriatal sensitivity to positive feedback and nucleus accumbens/ventral striatal hypoactivation [16]. This issue is compounded in biomarker studies that use syndromal severity scale total scores as primary outcomes, as total scores rarely reflect nuanced patterns of individual symptomatology. Additionally, there is no specific brain region or network functionally relevant for all 9 symptom dimensions captured in the DSM nosology, and hence correlations of biological and clinical measures will necessarily be plagued by low signal-to-noise scenarios. Strategies to contend with this problem have generally used data-driven approaches to either cluster patients into more homogenous biotypes on the basis of neuroimaging or genetic features[17, 18] or to identify homogenous symptom dimensions to model as primary outcomes rather than total scores.

Using this latter strategy, we have previously shown that neuroimaging biomarkers of antidepressant outcomes in electroconvulsive therapy (ECT) and serial ketamine infusion (SKI) can be improved by modeling dimensional components of depressive symptoms derived by applying exploratory factor analysis (EFA) to the 17-item Hamilton Depression Rating Scale (HDRS). Specifically, the EFA model identified dimensions of core mood and anhedonia (CMA), somatic disturbances (SoD), and insomnia [19, 20]. Here, we used machine learning to predict changes in depressive symptoms captured by the HDRS-17 and HDRS-6, as well as the CMA, SoD, insomnia subscales of the HDRS-17, and, as a secondary outcome, the Snaith-Hamilton Pleasure Scale (SHAPS) following rTMS using pretreatment RSFC measures as predictive features. We anticipated that the change in dimensional symptoms of the HDRS would be predicted more accurately than changes in the HDRS-17 total score, as we observed in ECT and SKI studies. We expected that pretreatment connectivity of the DMN and frontoparietal control network (FPCN) would be particularly predictive of treatment outcomes across scales and subscales and that outcomes would be predicted by overlapping but unique patterns of RSFC.

## Materials and Methods

### Participants

Twenty-six patients experiencing a depressive episode (mean age = 41.1±14.1 years, 50% male) diagnosed by the Structured Clinical Interview for DSM-IV were assessed between February, 2015 and November, 2020. Patient clinical and demographic characteristics are outlined in Table 1. All patients provided written informed consent and the study procedure was approved by the local ethics committee of the Massachusetts General Hospital.

Study inclusion criteria included that patients 1) were between the ages of 18–80 years old, 2) had a current DSM-IV diagnosis of a depressive episode, and 3) required TMS as part of their treatment for MDD. Exclusionary criteria included comorbid diagnoses of schizoaffective disorder, schizophrenia or dementia, substance use disorder, other severe medical illness, or contraindications to MRI or TMS.

### Treatment

Patients received rTMS to the left DLPFC (n = 17), right DLPFC (n = 5), or bilateral DLPFC (n = 4). Stimuli were delivered at 120% resting motor threshold at 10Hz to the left DLPFPC (3000 stimuli per session) or 1Hz to the right DLPFC (1800 stimuli per session). Stimulation was delivered at 110% resting motor threshold for one patient receiving 1Hz stimulation to the right DLPFC due to poor tolerability. The number of rTMS sessions varied as outlined in Table 1. Depressive symptoms were assessed using the HDRS-17 the day of their first and final treatment. Similarly, patients underwent resting-state fMRI within one week prior to their first treatment.

### Image Acquisition and Processing

Images were collected on a 3-Tesla Siemens Skyra MRI scanner (Siemens Healthcare, Malvern, PA, USA). Structural data was acquired with an anatomical T1-weighted multi-echo magnetization prepared rapid gradient-echo sequence with parameters: Repetition time (TR) = 2530ms, echo time (TE) = 1.69ms, inversion time (TI) = 1100ms, flip angle = 7.0°, number of excitations = 1, slice thickness = 1mm, field of view (FoV) = 256mm, in-plane resolution = 1.0 x 1.0mm, and a matrix of 256x256. Resting-state BOLD data was collected with a whole-brain echoplanar imaging sequence with the following parameters: TR = 3000ms, TE = 30ms, flip angle = 85°, slice thickness = 3.0mm, in-plane resolution = 3.0x3.0mm, FoV = 216mm. Subjects were instructed to keep eyes open and try to stay focused passively on a fixation cross displayed behind them during the resting-state scan (white cross on black background).

FMRI data were preprocessed using fMRIprep software package v. 20.2.1 [21]. Anatomical images were corrected for intensity bias with Advanced Normalization Tools N4BiasFieldCorrection (ANTs v. 2.3.3) (22). Images were segmented across tissue types in both ANTs and Freesurfer, and the previously estimated brain mask was refined by reconciling these two tissue segmentations using Mindboggle [23]. The T1 images were warped to MNI space using nonlinear registration with antsRegistration (ANTs 2.3.3).

Functional images were skull-stripped and aligned to the anatomical reference image using boundary-based registration with six degrees of freedom in Freesurfer. Functional images were then slice-time corrected (AFNI v. 201660207)[24] and motion-corrected (FSL v. 5.0.9) [25]. These images were then normalized to MNI space, using the warp previously computed for transforming the T1 image to MNI space. Fmriprep’s implementation of anatomical *CompCor*[26] was used to estimate principle components for signal originating in white matter and CSF, respectively. The data were then blurred using a 5mm smoothing kernel and submitted to confound regression using nilearn.glm.first_level. This regression included regressors for six motion parameters and their derivatives, and enough aCompCor principle components to capture 50% of the variance of signal from white matter and CSF, respectively (a maximum of five components). Regressors were also added to scrub the first 3 TRs and high motion TRs. High motion TRs were defined as those having a framewise displacement over .3 mm. Runs with greater than 50% of TRs meeting this threshold were excluded from analysis. Sessions with fewer the 5 minutes of data after censoring were excluded.

### Predictive Features

Pretreatment rs-fMRI scans were parcellated using multiple atlases. We included 200 cortical regions using the Schaefer atlas [27], 24 regional parcellations from the Kelly insula atlas [28], 21 subcortical parcellations from the Harvard-Oxford Atlas [29], 16 nuclei from the Pauli subcortical atlas [30], 10 parcellations from the Pauli amygdala nuclei atlas [31], the bilateral bed nucleus of the stria terminalis, and the grey matter of the TMS target for 274 regional measures. Supplementary Table 2 lists each predictor. Global connectivity (GC) values for each ROI were computed subjectwise using the graph theoretic node degree as previously reported in [20]. NiLearn [32] scripts were used to compute correlations of time series data between each ROI. Subjectwise correlation matrices were thresholded at Z≥∣0.4∣ to create a binary adjacency matrix. The graph theoretic node degree for each parcellation was computed as the number of other regions with which the given region was correlated above the threshold of Z≥∣0.4∣, serving as a proxy of GC for each ROI.

### Outcome Measures

Primary outcomes included changes along the depression severity HDRS-17 total score [33] and the HDRS-6 subscale [34]. We also included a previously identified three-factor solution [19, 35] for the HDRS-17 which captures dimensions of core mood and anhedonia (CMA), somatic disturbances (SoD), and insomnia. Items of the HDRS composing each subscale are outlined in Supplementary Table 1. As a secondary outcome, we directly investigated changes in hedonic tone using the Snaith-Hamilton Pleasure Scale (SHAPS) [36].

### Predictive Modeling

Random forest regression (RFR) models were trained to predict change in each outcome. Models were trained and tested using 10-repeated 10-fold cross validation with a nested grid search for parameter optimization. The parameter grid tuned the number of features selected, k, using mutual information regression with k={10,50,100,p}, where p is the number of all predictors; number of regression trees n_tree={50,100,500,1000}. Model performance was evaluated using the sums of squares formulation of $R^{2}$; i.e., $1-\frac{\sum_{i}(y_{i}-{\hat{y}}_{i}{)}^{2}}{\sum_{i}{\left(y_{i}-\bar{y}\right)}^{2}}$, where ${\hat{y}}_{i}$, is the predicted outcome of the i-th subject and ${\bar{y}}_{i}$ the average outcome across all subjects in the testing fold [37]. The significance of model performance was tested using permutation tests with B = 100 permutations. Model significance was adjusted for multiple comparisons across primary outcomes using a Bonferroni correction, yielding a critical value of 0.05/5 = 0.01. Models were constructed using Scikit-learn v1.1.0 [38].

### Post-hoc Analysis

Components of the DMN and dorsal attention network (DAN) were predictive of CMA, HDRS-17, and HDRS-6 outcomes and the activity of both networks have been associated with ruminative symptoms [39-41]. Therefore, as post-hoc analyses, we conducted Pearson correlation tests to determine whether the pretreatment Rumination Response Scale (RRS) [42] total score and brooding and reflection subscales were associated with (1) changes in the CMA, HDRS-17, and HDRS-6 scales, and (2) with the top six most predictive GC measures across those outcomes.

## Results

### Cohort Characteristics

With treatment response defined as ≥50% reduction in the HDRS-17 total score, 42% of the cohort were treatment responders. Female patients experienced greater symptom reduction than males (t = 2.10, p < 0.05). Patient age was not significantly associated with the degree of HDRS-17 symptom reduction. The degree of symptom reduction across all outcomes is reported in Table 1.

### Model Performance

**Figure 1:**
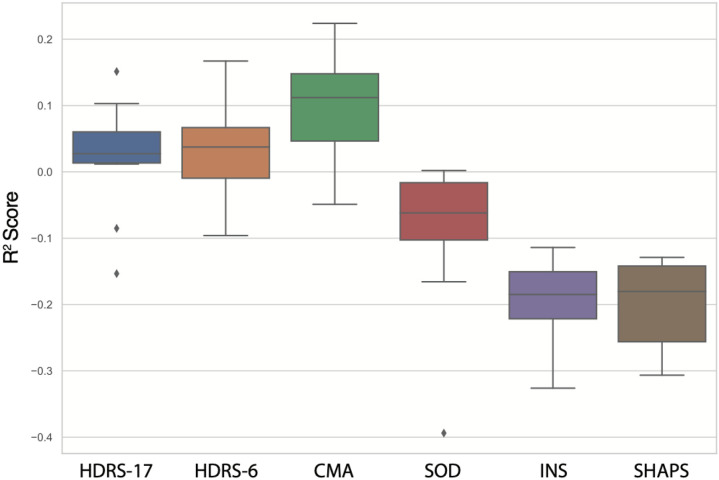
Boxplots illustrating the distribution of R^2^ scores (i.e., the fraction of explained variance) for primary and secondary outcomes across repeated cross validation folds in held-out data.

Change along the CMA dimension was predicted significantly above chance ($R^{2}=0.09$, p < 0.01). The HDRS-6 subscale ($R^{2}=0.02$, p < 0.01) and HDRS-17 total scores ($R^{2}=0.02$, p < 0.01) were also predicted significantly. The mean $R^{2}$ for the CMA outcome was significantly higher than the HDRS-17 (p < 0.05). The remaining primary and secondary outcomes (SoD, insomnia, and SHAPS) were not predicted significantly (all p > 0.05). Model performance is outlined in Table 2 and $R^{2}$ distributions are illustrated in Fig. 1.

### Connectivity Patterns Predictive of Change in Core Mood & Anhedonia

**Figure 2:**
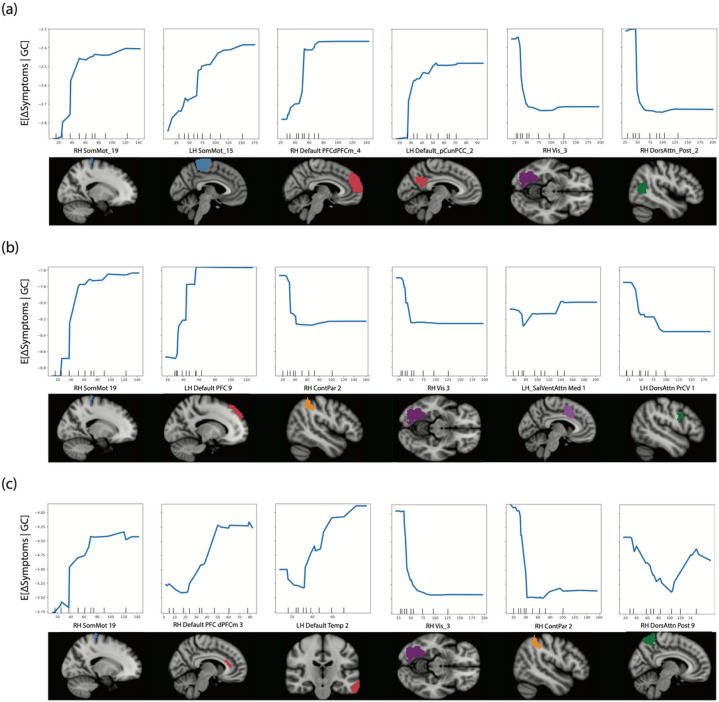
Partial dependence plots for significantly predicted outcomes illustrating the expected change in symptoms (y-axis) across the observed range of the top six imaging predictors (x-axis). We also highlight the Schaefer atlas parcellations with predictive global connectivity measures below each plot. Section (a) highlights partial dependence plots for the core mood/anhedonia (CMA) dimension. From left to right RH_SomMot_19 (right dorsal primary motor cortex); LH_SomMot_15 (left dorsomedial primary somatosensory cortex), RH_Default_PFCdPFCm_4 (right anterior medial PFC), LH_Default_pCunPCC_2 (precuneus/posterior cingulate cortex), RH_Vis_3 (right fusiform area), RH_DorsAttn_Post_2 (right extrastriate cortex). Section (b) partial dependence plots for 17-item Hamilton Depression Rating Scale (HDRS). From left to right, predictors are RH_SomMot_19 (right dorsal primary motor cortex), LH_Default_PFC_9 (left dorsomedial prefrontal cortex), RH_ContPar_2 (right supramarginal gyrus), RH_Vis_3 (right fusiform area), LH_SalVentAttn_1 (left dorsal anterior cingulate), and LH_DorsAttn_PrCV_1(left premotor/supplementary motor cortex). Section (c) illustrates partial dependence and regional predictors for the HDRS-6 outcome. From left to right, predictors are RH_SomMot_19 (right dorsal primary motor cortex), RH_Default_PFC_dPFCm_3 (dorsomedial PFC/ventral anterior cingulate), LH_Default_Temp_2 (left medial temporal gyrus), RH_Vis_3 (right fusiform area), RH_ContPar_2 (right supramarginal gyrus), and the RH_DorsAttn_Post-9 (right precuneus).

Increased GC of regions comprising the right dorsal primary motor cortex within Brodmann Area (BA) 4 and left dorsomedial primary somatosensory cortex in BA1 (both components of the somatomotor network [SMN]), and right anterior medial PFC (mPFC) in BA10 and left precuneus (pCun)/posterior cingulate cortex (PCC) in BA23 within the DMN was predictive of poorer reduction of CMA symptoms. Conversely, increased GC of parcels within the right extrastriate cortex in BA19 of the DAN and right fusiform gyrus in BA37 of the visual network (VN) predicted greater reductions in CMA symptoms. Figure 2(a) illustrates regional parcellations predictive of change for the CMA outcome. Table 3 outlines characteristics of regional predictors for each significantly predicted outcome.

### Connectivity Patterns Predictive of Change in the HDRS-17 and HDRS-6

Increased pretreatment GC of the right dorsal primary motor cortex (BA4) within the SMN and left dorsomedial PFC (BA8) within the DMN predicted poorer reduction of HDRS-17 symptoms which was similar to what was observed for the CMA dimension. Increased GC of the right supramarginal gyrus (BA40) FPCN, the right fusiform gyrus (BA37) within VN, and left premotor/supplementary motor cortex (BA6) within the DAN predicted greater reductions in HDRS-17 symptoms. Change in the HDRS-6 subscale was predicted by similar regional connectivity patterns: increased GC of right dorsal primary motor cortex (BA4) within the SMN, and the left medial temporal gyrus (BA21) and right dmPFC/ventral anterior cingulate (BA24) within the DMN predicted poorer symptom reduction. Increased GC of the right supramarginal gyrus (BA40) within the FPCN and right fusiform gyrus (BA37) within VN predicted greater reductions in symptoms. Increased GC of the right parietal precuneus BA7 within the DAN predicted a U-shaped change in symptoms, with low and high GC values predicting higher symptoms and mid-range GC values predicting decreased symptoms. Figure 2(b-c) highlights regional parcellations predictive of change in the HDRS-17 and HDRS-6 outcomes.

### Post-hoc Evaluation of Ruminative Symptoms

Pearson correlation tests identified no associations between the pretreatment RRS total score, Brooding, or Reflection subscales and changes in the CMA, HDRS-17, or HDRS-6 scales or GC measures predictive of changes in those outcomes (all p > 0.05).

## Discussion

We investigated whether dimensional changes in depressive symptoms following rTMS were predictable using multivariate patterns of pretreatment RSFC. Changes along the CMA dimension and HDRS-17 and HDRS-6 scores were predicted significantly and our findings are convergent with earlier studies in ECT and SKI which suggested that change in the CMA dimension is predicted more robustly than other symptom clusters. Specifically, changes in CMA were largely informed by pretreatment GC of key nodes within the DMN, SMN, DAN, and VN: higher pretreatment GC of the SMN (primary motor [BA4] and somatosensory cortex [BA1]) and DMN (mPFC [BA10] and precuneus/posterior cingulate [BA23]) predicted poor outcomes while higher GC of the VN (fusiform gyrus [BA37]) and DAN (extrastriate cortex [BA19]) predicted better outcomes. Changes in the HDRS-17 and HDRS-6 total scores were predicted by two GC patterns that overlapped with the CMA dimension: The right primary motor cortex (spanning BA4) and the right fusiform gyrus (spanning BA37). The GC of the right supramarginal gyrus (BA40) within the FPCN was a common predictor for both changes in the HDRS-17 and HDRS-6 outcomes. Beyond these, important predictors were unique to each outcome; however, unique parcellations within overlapping large-scale resting-state networks exhibited similar relationships with symptom changes across primary outcomes. For example, increased GC of the DMN and SMN predicted poorer symptom changes across all primary outcomes while increased GC of components within the DAN and VN generally predicted more symptom reduction.

### Dimensional clinical measures are predicted with greater accuracy than syndromal severity outcomes

A central aim in precision psychiatry is the identification of biomarkers predictive of individual outcomes following antidepressant treatment. Identification of such biomarkers is obscured by the symptomatic heterogeneity of depression and the use of scale total scores as model outcomes. Critically, distinct constellations of symptoms may be related to separable patterns of aberrant neural connectivity and indicate differing likelihoods of responding to a treatment. Thus, total score outcomes are prone to simply agglomerate multiple possibly separable syndromal dimensions and fail to reflect potentially more nuanced and clinically relevant aspects of an individual’s symptomatology. The framework we have introduced in previous studies on ECT and SKI [19, 20] and applied here with rTMS, is one means by which to contend with symptom heterogeneity and these findings collectively support that modeling homogenous symptom dimensions may improve prediction of treatment outcomes.

### Functional neuroanatomy and clinical implications

Here, we will outline the potential clinical significance of the more informative predictors and their related circuitry in predicting antidepressant outcomes following rTMS.

### Default Mode Network (DMN)

The DMN is often conceptualized as a network of three subsystems [43]: a midline core encompassing the PCC and anterior mPFC, a dmPFC subsystem which also includes temporal regions [44, 45], and a medial temporal lobe system. Activity within the DMN has been related to self-referential thought facilitated by hippocampo-cortical connectivity [46]. Dysfunctional DMN connectivity has been associated with maladaptive ruminative symptoms[39, 40] and emotion dysregulation[47] which, in turn, is predictive of treatment resistance [48]. Echoing this, we observed that elevated pretreatment connectivity of the dmPFC and pCun/PCC, key nodes in two DMN subsystems, predicted treatment resistance.

The dmPFC has been implicated in roles of emotional and behavioral regulation [49-52]. Given its involvement in these domains, the dmPFC has also been used as a potential TMS target site for a variety of disorders including depression [49, 53], obsessive-compulsive disorder [54], and borderline personality disorder [55]. Anterior components of the DMN (aDMN) are hyperconnected in depressed patients [56-58] and this hyperconnectivity may be predictive of antidepressant response following rTMS [59]. An earlier study of ours reported that the aDMN was predictive of CMA improvement following SKI treatment [20]. In the SKI study, however, increased connectivity of the aDMN was associated with greater therapeutic response in CMA symptoms. This discrepancy may relate to differences in treatment mechanisms, in line with recent studies that identified differential biological mechanisms of established interventional antidepressants, i.e. ECT and TMS [60]. Alternatively, slight variations in boundaries of aDMN components may partially account for this as a different atlas was used in our previous study.

The PCC is a central node in the posterior DMN [61] with functional roles in internally directed cognition [62] and guiding attention [63]. It is a highly metabolically active and broadly interconnected region [64] with PCC subregions being functionally connected to other large-scale resting-state networks including the salience network, DAN, and SMN [64-66]. Disrupted static [67] and dynamic [68] functional connectivity between the PCC and the aDMN has been reported in depression with the degree of disruption associated with ruminative symptoms [69]. One study [70] reported that the PCC’s connectivity is elevated in depression while Blum et al. reported reduced connectivity between the PCC and the caudate in unmedicated patients [71]. The previously mentioned SKI study from our group using a similar predictive framework identified that increased pretreatment functional connectivity of the PCC (BA v23ab) predicted less reduction of ruminative symptoms captured by the RRS Reflection subscale [42] which parallels our findings of increased PCC GC predicting refractory symptoms.

### Somatomotor Network (SMN)

Higher pretreatment SMN connectivity also predicted refractory CMA symptoms. A large study of 848 patients with MDD and 794 unaffected controls reported reduced within network connectivity of the SMN and disrupted SMN-DAN connectivity in depressed participants [72]. Another study in adolescents with depression reported similar patterns of hypoconnectivity of the SMN relative to controls [73]. The RSFC of the SMN has also been identified in predictive studies. For example, in a cohort of 163 patients with depression receiving escitalopram, sertraline or venlafaxine-XR, greater connectivity between the SMN-DMN as well as the SMN with cingulo-opercular network and DAN was predictive of remission after eight weeks of treatment [74]. A study by Leaver et al. also reported that pretreatment RSFC of components of the SMN were predictive of antidepressant response to ECT [75].

### Dorsal Attention Network (DAN)

The DAN is involved in external orientation of attention and has also been implicated in the pathophysiology of depression. A large meta-analysis involving 556 patients with depression and 518 controls noted widespread hypoconnectivity between the DAN and the FPCN[41] in patients with depression. A study using both task-based and resting-state functional connectivity identified altered low-frequency oscillations of the DAN in unmedicated patients with depression [76]. Disrupted DAN connectivity has been hypothesized to be a signature of attentional bias towards internally-oriented attention (e.g., rumination) at the expense of externally-oriented attention [41]. In contrast with the DMN, we observed that increased pretreatment GC of regions within the DAN predicted more reduced symptoms across the CMA, HDRS-17, and HDRS-6 scales. Taken together with our DMN findings, we hypothesized that connectivity patterns that were predictive for this set of outcomes may be related to the severity of ruminative symptoms which might also be related to subsequent symptom changes; that is, symptoms of rumination might mediate associations between regional connectivity and symptom reduction. Exploring this, however, we observed no evidence for these associations.

### Visual Network (VN)

Higher pretreatment connectivity of the right fusiform area, a component of the visual network, was predictive of more reduced symptoms following rTMS. Perceptual processing deficits have previously been reported in MDD. For example, eye tracking studies report that patients with depression disproportionately gaze at dysphoric stimuli relative to controls [77] and perceptual processing deficits for non-aversive stimuli have been reported in depressed patients [78]. Several studies have further reported specific functional connectivity abnormalities of the fusiform area in depression. A study of 62 patients with depression and 61 controls identified disrupted connectivity between the fusiform area and sensorimotor regions of the pre- and post-central gyrus [79] while a separate task-based study in 26 adolescences with depression and 37 matched controls using an affective face processing paradigm reported reduced fMRI signal in the left fusiform gyrus during facial emotion identification compared to controls [80]. Treatment-related effects of ketamine have been shown to modulate aberrant connectivity of the broader visual network in depressed patients [81]. Task and resting-state fMRI measures of the visual network have also been identified as candidate predictors of antidepressant treatment response for electroconvulsive therapy [82] and cognitive behavioral therapy [83].

### Fronto-parietal control network (FPCN)

Higher connectivity of the right supramarginal gyrus (BA40), a component of the FPCN, was predictive of more reduced symptoms following rTMS. The FPCN serves as a “functional hub” that modulates communication across other brain networks to flexibly meet task-oriented demands [84] and has been linked with transdiagnostic symptom disturbances [85] including depression [86]. In line with this and our own findings, a recent fMRI study reported that decreased GC of the FPCN with the rest of the brain was associated with more severe depressive symptoms in the general population [87]. Taken together with previous investigations, our findings may reflect that patients with more intact cognitive control are more responsive to rTMS.

### Limitations

There are several limitations to consider. Notably, this is a small cohort and the number of predictive features is much larger than the number of subjects which leaves models more prone to overfitting. To combat this, however, we used a conservative 10-repeated 10-fold cross validation approach wherein model performance is evaluated on held-out data and performance is aggregated across repeated cross validations. Furthermore, TMS parameters were not uniform across participants; however, this reflects the natural variation seen in clinical practice.

## Conclusions

We used a data-driven approach to identify candidate biomarkers of dimensional symptom changes following rTMS. Importantly, these findings converge with two earlier studies in electroconvulsive therapy and serial ketamine infusion wherein the degree of change in CMA symptoms was predicted more accurately than broader changes of the HDRS-17 and HDRS-6, highlighting the importance of dimensional measures in therapeutic studies and biomarker development research. Notably, the patterns of pretreatment connectivity informing symptom change are in networks and regions affected by the pathophysiology of depression: anterior and posterior components of the DMN, components of the DAN, VN, and the SMN. We observed that increased connectivity of the DMN and SMN robustly predicted more treatment resistance while increased connectivity of the DAN, FPCN, and VN largely predicted higher therapeutic efficacy. This work may help us to characterize patients who are well- or poorly indicated for rTMS and further highlights the importance of adopting dimensional approaches to modeling outcomes as a viable strategy to accelerate the discovery of biomarkers for treatment stratification.

## Figures and Tables

**Table 1:** Demographic and clinical information

N	26
Age, mean (SD)*	41.1 (14.1)
Sex, male/female	13/13
*Clinical Rating Scales*	
HDRS-17 baseline, mean (SD)	18.3 (5.5)
HDRS-17 percent change, mean (SD)	−41.6% (33%)
HDRS-6 baseline, mean (SD)	10.0 (2.4)
HDRS-6 percent change, mean (SD)	−41.2% (36%)
CMA baseline, mean (SD)	5.5 (1.9)
CMA percent change, mean (SD)	−37.6% (51%)
SoD baseline, mean (SD)	8.8 (3.3)
SoD percent change, mean (SD)	−39.8% (44%)
INS baseline, mean (SD)	2.5 (1.7)
INS percent change, mean (SD)	−41.1% (45%)
SHAPS baseline, mean (SD)	6.2 (3.4)
SHAPS percent change, mean (SD)	−28.8% (122%)
HDRS-17 Responder/Non-Responder, N	11/15
HDRS-17 Remitter/Non-Remitter, N	9/17
*Treatment Parameters*	
Left DLPFC Target, N*	17
Right DLPFC Target, N	5
Bilateral DLPFC Target, N	3
Number of sessions, #Sessions (N)	30 (n = 3)
	36 (n = 18)
	71 (n = 1)
	72 (n = 4)
10 Hz – 3000 pulses, N*	19
1 Hz – 1800 pulses, N	6

* Value missing for one participant

Abbreviations: HDRS: Hamilton Depression Rating Scale; CMA: Core Mood/Anhedonia; SoD: Somatic Distrubances; INS: Insomnia; SHAPS: Snaith-Hamilton Pleasure Scale; DLPFC: Dorsolateral Prefrontal Cortex

**Table 2:** Model Performance

Outcome	Mean \$\text{varvec}R^{2}$
HDRS-17	0.020**
HDRS-6	0.027**
CMA	0.099**
SOD	−0.092
INS	−0.197
SHAPS	−0.198

** Significant at the p < 0.01 level

Abbreviations: HDRS: Hamilton Depression Rating Scale; CMA: Core mood and anhedonia; SOD: Somatic disturbances; INS: insomnia

**Table 3.** Outline of regions predictive of outcomes

Core Mood and Anhedonia					
Schaefer 200 Atlas Label	RAS Centroids	Yeo 7 Network	Brodmann Area	Region	Relationship to Outcome
RH_SomMot_19	20, −30, 70	SMN	BA 4	dorsal primary motor cortex	+
LH_SomMot_15	28, −34, 66	SMN	BA 1	dorsomedial primary somatosensory cortex	+
RH_Default_PFCdPFCm_4	8, 58, 18	DMN	BA 10	anterior medial PFC	+
LH_Default_pCunPCC_2	−6, −54, 28	DMN	BA 23	precuneus/posterior cingulate cortex	+
RH_Vis_3	28, −68, −12	VIS	BA 37	fusiform area	−
RH_DorsAttn_Post_2	52, −60, 10	DAN	BA 19	extrastriate cortex	−
HDRS-17					
RH_SomMot_19	20, −30, 70	SMN	BA 4	dorsal primary motor cortex	+
LH_Default_PFC_9	−12, 48, 44	DMN	BA 8	dorsomedial PFC	+
RH_Cont_Par_2	52, −42, 48	FPCN	BA 40	supramarginal gyrus	−
RH_Vis_3	28, −68, −12	VIS	BA 37	fusiform area	−
LH_SalVentAttn_Med_1	−6, 10, 42	VAN	BA 32	dorsal anterior cingulate	mixed
LH_DorsAttn_PrCv_1	−48, 6, 28	DAN	BA 6	premotor/supplementary motor cortex	−
HDRS-6					
RH_SomMot_19	20, −30, 70	SMN	BA 4	dorsal primary motor cortex	+
RH_Default_PFCdPFCm_3	6, 28, 16	DMN	BA 24	dorsomedial PFC/ventral anterior cingulate	+
LH_Default_Temp_2	−60, −18, −22	DMN	BA 21	medial temporal gyrus	+
RH_Vis_3	28, −68, −12	VIS	BA 37	fusiform area	−
RH_Cont_Par_2	52, −42, 48	FPCN	BA 40	supramarginal gyrus	−
RH_DorsAttn_Post_9	8, −56, 62	DAN	BA 7	precuneus	mixed

Abbreviations: SMN: Somatomotor Network; DMN: Default Mode Network; VIS: Visual Network; DAN: Dorsal Attention Network; FPCN: Frontoparietal Control Network; VAN: Ventral Attention Network; BA: Brodmann Area; HDRS: Hamilton Depression Rating Scale
